# “We missed the psychological support”: A case study about the preparation of the Brazilian bronze medal kata team for the 2019 Pan American Games

**DOI:** 10.3389/fpsyg.2022.1074357

**Published:** 2023-01-12

**Authors:** Ana Carolina Paludo, Cintia Lassalvia, Iryna Mazhak, Jan Cacek, Danilo Fernandes da Silva

**Affiliations:** ^1^Incubator of Kinanthropology Research, Faculty of Sports Studies, Masaryk University, Brno, Czechia; ^2^School of Physical Education and Sport, University of São Paulo, São Paulo, Brazil; ^3^Department of Athletics, Swimming and Outdoor Sports, Faculty of Sports Studies, Masaryk University, Brno, Czechia; ^4^Department of Sports Studies, Faculty of Arts and Science, Bishop’s University, Sherbrooke, QC, Canada

**Keywords:** qualitative, karate, kata, Pan American Games, training

## Abstract

**Purpose:**

The main aim of the study was to describe the key factors involved in the preparation process of the Brazilian bronze medal kata team for the 2019 Pan American Games, focusing on the athletes’ perceptions.

**Methods:**

Three male athletes from the Brazilian team performed a semistructured interview to identify the following topics: specific time for preparation, training organization, supplementary support, and perception and suggestion about the efficiency of the preparation process.

**Results:**

Data from interviews were gathered and coded, and the major themes were summarized as follows after performing content analysis of the data: (a) technical and tactical training took the major part of the preparation; (b) the high level of the coaches helped the team to reach the technical quality of the kata; (c) better psychological support during the preparation could improve the athletes’ performance during the training and competition; and (d) the lack of financial support compromised the commitment of the athletes during the training routine.

**Conclusion:**

We concluded, based on the athletes’ perception, that the most positive factor during preparation for a major competition was the high amount of time focused on technical-tactical training. Even with limitations to performing the physical training, the athletes recognized the importance of the physical component, to increase performance. Financial and psychological support could have helped the team to reach a better result (gold medal) attenuating the training distress.

## Introduction

Karate is an ancient martial art that originated on the island of Okinawa, South of Japan, and was recognized as a martial art at the beginning of the 20th century (Arriaza, 2009). Besides the martial art characteristics, karate is also a competitive sport modality, organized by the World Karate Federation (WKF) since 1970, a maximal organization related to the International Olympic Committee. Karate can be divided into two categories: Kumite and Kata. Kumite is a technical combat category between two athletes characterized by high-intensity 3-min bouts involving kicking, punching, and quick horizontal displacements with no knockout allowed. The winning is based on scores obtained according to correct techniques applied against the opponent. Kumite athletes can compete individually and in teams of both sexes (World Karate Federation [WKF], 2017; Chaabene et al., 2019). Kata competition is described as an intermittent demonstration of offensive and defensive karate techniques that represents actual fights against fictitious opponents. Kata athletes (men or women) can compete either individually or synchronously in a team (three athletes per team) (World Karate Federation [WKF], 2017). Moreover, kata was created as a karate exercise in order to develop coordination and rhythm of the movements, to improve the karateka technique (Nakayama, 1973) and mental aspects such as concentration, focus, and discipline (Funakosh, 2014).

In the kata competition, seven referees evaluate the athlete’s presentation considering athletic and technical criteria. Athletic criteria are based on strength, speed, and balance, and technical criteria consider the stances (i.e., leg postures), basic techniques, transitional movements, timing, correct breathing, focus (“*kime*”), and compliance with the style. There are four different styles called *Shotokan*, *Shito-ryu*, *Goju-ryu*, and *Wado-ryu* recognized by the WKF which compete together during official competitions. To compose the presentation, the athletes must perform one of the 102 kata from a WKF list (World Karate Federation [WKF], 2017). Usually, the most common kata performed in competitions lasts between 90 and 180 s (Lassalvia et al., 2021) and is chosen by the athletes according to their experience, level of difficulty, and opponent level (Augustovicova et al., 2018).

In high-level competitions, professional kata athletes usually have a long-term career presenting elevated technical-tactical skills and physical fitness components due to a higher amount of training hours. During the process of preparing for a competition, training is one of the main aspects necessary to achieve a successful result, together with complementary strategies for athletic health management (e.g., nutrition, physical therapy, and psychological support) (Issurin, 2010; McGuigan, 2017; Bompa and Buzzichelli, 2019). The complex combination of training factors used during the preparation process can play a key role in the success of the athlete in the competition (Bompa and Buzzichelli, 2019). However, this information in the kata discipline has yet to be described. Considering that the kata discipline is not frequent in the Olympic Games, continental competitions, such as Pan American in the American continent, represent one of the major international events in which a Brazilian kata athlete can compete. Therefore, understanding the preparation of kata athletes for this outstanding competition can bring important information to researchers, coaches, and athletes, given that the evidence in this sports discipline is still scarce. This study aimed to describe the key factors involved in the preparation process of the Brazilian bronze medal kata team for the 2019 Pan American Games, focusing on the athletes’ perceptions.

## Materials and methods

The case study utilized a qualitative approach, based on a semistructured interview to provide information about the phenomenon of interest. The case study approach allows for a better understanding of the athletes’ experiences within a real-life context and involved with specific circumstances or incidents. However, case studies have limited generalizability (Stake, 2000; Patton, 2002). Moreover, the interview approach is often aimed at obtaining information or studying a particular field to answer the research question. Some studies in the field of exercise and sports medicine need to measure behavioral perspectives, such as athletes’ motivation, attitudes, beliefs, and perceptions. Usually, these factors are difficult to measure without using qualitative research methods such as interviews. Qualitative methods are also useful in the study of contextual factors that affect physical activity and athletic performance (Draper, 2009). In the present study, the experts (i.e., kata athletes) are the key informants who have specific knowledge and skills, and for that reason, they become a source of information. Therefore, semistructured questions have been designed to explore the preparation process used by a bronze-medal Brazilian male kata team in the 2019 Pan American Games with open-ended questions to understand the key factors perceived by the athletes.

### Participants

The participants consisted of three kata athletes that represented the Brazilian male team in the 2019 Pan American Games, winning a bronze medal (Table 1). The team was previously qualified due to succeeding in a specific competition to represent the team Brazil in the Pan American Games. The 2019 Pan American Games was held in Lima, Peru, with about 6,700 athletes participating in 39 sports and 61 disciplines. Twenty-two of these sports were qualifiers for the Tokyo 2020 Olympic Games. Kata competition occurred as a subdiscipline of karate, with individual and team competitions (LIMA, 2019). The study was reviewed and approved by the Ethics Committee from UNICENTRO University (Protocol 20449319.4.0000.0106), and the athletes signed the consent form before data collection.

**TABLE 1:** Athletes’ description during the 2019 Pan American Games.

Code	Age (years)	Time training kata (years)	Time competing at a professional level (years)
Athlete 1 (A1)	25	15	10
Athlete 2 (A2)	19	10	8
Athlete 3 (A3)	25	20	15

### Script of interview

Semistructured interviews were used to obtain an understanding of athletes’ perceptions of the 2019 Pan American Games preparation. The questions were developed by three researchers: one researcher and former athlete of kata; one researcher with previous experience in qualitative design (e.g., semistructured interview) and training monitoring and prescription; and one researcher with experience in the training program who had contact with the athletes. The three researchers agreed to separate the questions into four categories for the analysis: (i) specific preparation for the 2019 Pan American Games; (ii) training organization; (iii) complementary support; and (iv) perception and suggestions about the efficiency of the training plan. At the end of the interview, athletes were asked “*Is there any question that was not asked, and you believe it should have been?*” (Table 2).

**TABLE 2:** Structured interview questions.

Questions and prompts
**Specific preparation for the 2019 Pan American Games**
1. When did the team start the preparation for the qualifiers to compete in the 2019 Pan American Games?
2. When you learned that your team had been classified to compete in the 2019 Pan American Games, have you and/or your team thought about doing something different from what you were already doing to achieve a medal?
**Training organization**
3. Did you organize the training based on the technical scoring criteria to score higher in the competition?
4. Can you describe how you distribute the technical-tactical and physical components in the training preparation? E.g., per day, per period of the day (morning/afternoon), per week?
5. Can you rate the importance of the technical-tactical and physical components in your preparation?
6. Did you have any professional support during the training period? What was their role (i.e., technical-tactical or physical component)?
7. Did you have any conversation with your team or coach about the training efficiency? Did the training work as planned?
**Complementary support**
8. Did you have support for any professional in addition to training (e.g., physical therapist, nutritionist, psychologist)?
9. Did you have sponsors during the preparation period?
**Training perception and further suggestions**
10. Looking back at the preparatory period, what do you think that worked well and what would you do differently?
11. If you had to start a new training plan for an international championship, how would you structure it?
Is there any question that was not asked, and you believe it should had been? Is there any aspect of your training that you would like to highlight about the preparation period?

### Interviewer and interview procedure

The three interviews were conducted by one researcher who had previous contact with the athletes, which allowed them to guide the interview using specific terminology associated with the training program and generate a comfortable environment with the interviewees. Interviews with experts were conducted online. More specifically, *Google meeting*^®^ platform was used for the interviews to enable visual contact (i.e., video was kept on). Interviews lasted between 28 and 35 min and were recorded to be subsequently transcribed. The analysis and transcribed interviews were based on thematic coding to identify themes in qualitative data (Given, 2008). Then thematic analysis was performed according to what was proposed by Braun and Clarke (2021) and outlined in more detail under section “Data analysis.” Transcripts were coded by two researchers who met after this process to discuss themes based on the highlighted codes. Instead of using a statistical quantifiable procedure to assess inter-coder reliability (e.g., percentage agreement, Cohen’s Kappa, Krippendorff’s Alpha), we opted to have researchers meeting and presenting to each other their views and interpretations with the ultimate goal to come up with a consensus of the main codes/themes. This procedure allows researchers’ multiple perspectives of reality to be communicated and interpreted, and respects the diversity of their social context and personal history (Bauer et al., 2000). This approach also maximizes researchers’ reflexivity and self-engagement with the qualitative nature of the research project (Yardley, 2008).

### Data analysis

Qualitative data was based on content analysis, using six steps process for data engagement, coding, and theme development (Braun and Clarke, 2021): (i) data familiarization (reading and rereading the transcriptions of interviews); (ii) systematic data coding (coding the whole texts and identify the part of the texts which are answering the research questions); (iii) generating initial themes from coded and collated data (combined the data into four main themes); (iv) developing and reviewing themes (detailed analysis of the themes); (v) refining, defining and naming themes (name, split, combined, or discarded themes, confirming if research questions were answered); and (vi) writing the report.

The transcribed texts were analyzed using the qualitative data analysis software NVivo version 12 (QSR International Pty Ltd, 2018), and the transcription was made by the same researcher who performed the interviews and revised by a second researcher. Two researchers analyzed the verbal reports independently, and subsequently, the researchers determined themes and subthemes in each category. Themes are generally phrases or sentences that describe more complex and latent processes across the cases in the study (Saldana, 2013; Connelly and Peltzer, 2016).

## Results

After the interview, the data was transcripted and coded, and the findings were expressed in themes and subthemes for each topic, described as follows:

### Specific preparation for the 2019 Pan American Games

The athletes reported that the team was created approximately 1 year prior to the competitions to allow adequate preparation for the qualifiers of the 2019 Pan American Games. It was the first time that the team was put together with this particular formation (i.e., three athletes together). After the qualifying competition for the 2019 Pan American Games, the athletes reported that the training plan was organized and focused on correcting the mistakes that happened in the previous qualifying competition. In addition, the focus was on improving their performance in order to exhibit different technical skills that could increase their score and reach the gold medal. “*As much as we already knew which teams we were going to compete with, it was possible to get a sense of what we were missing, what we needed to improve (A2)*.” “*We needed to improve physically and mentally (A3)*.”

### Training organization

At the beginning of the preparation, the coaches focused on the adaptation of the three athletes. “*In the beginning, it is about the adaptation of the three athletes (A3)*.” As the team had a new formation and a large age range (i.e., A2 was 19 and A3 25 years old), the preparation started with strengthening the relationship among the three athletes and understanding the strengths and limitations of each one. “*A kata team does not depend only on you (A3)*.” The choice of the kata performance was based on the best Kata performed by each athlete, in addition to what referees had previously scored the highest in other competitions. The physical performance component is prioritized in the male kata team, as well as the impact of each movement as they are executed. Therefore, “*the physical component was highly taken into consideration (A1)*.” However, the technical component scores 70% of the total score, thus, the emphasis on the technique was higher during the training preparation. “*Even if the athlete is thin, when they perform the movement correctly, they stand out for the referees (A2)*.”

Regarding the training organization, the athletes separated the training into two to three sessions per day, for physical and technical-tactical components. The training was performed every day, with only 1 day off (usually on Sundays). The physical component was trained individually and the technical-tactical sessions were performed by all athletes together with the coach (i.e., *sensei*). “*Each athlete chose the best schedule to do the physical training alone but the technical training should be done as a team (A3).” “We dedicated 30–40% of the total training time to perform a strength training program and the remaining was divided between ‘kihom’ and tactical training*.” Kihon is defined as one aspect of karate training and can be explained as technical training of all the strikes, stances, and displacements in order to improve the combination of power, balance, and coordination during the movements (Lassalvia et al., 2022). Two athletes had to work besides training (A1 and A2) and one athlete was an undergraduate student in Physical Education (A3). Considering their activities outside the training program, the physical sessions occurred according to the availability of each athlete. The athletes reported difficulties in fitting the physical sessions into their daily routines. “*I trained sometimes in the late evening or very early in the morning (A2)*.” However, they recognized that the physical sessions played an important role in the preparation process for the 2019 Pan American Games compared to previous competitions.

Most parts of the training were devoted to the technical movements (kata) and “*kihon*” movements. These training sessions were performed with the three athletes together or individually with the coaches (*sensei*). Athlete A2 reported training sometimes four times per day. In the few weeks immediately before the competition, the team focused on refining the movements (kata) and training every day with no day off. “*As the competition was getting close, our training was becoming more similar to the competition day, to be more realistic (A1).” “Our focus in technical training increased, since the three of us needed to perform exactly the same movement (A3)*.” The athletes described that the technical session began to get longer in duration. The movements were recorded and analyzed by all athletes together, in order to correct their detail. “*Focus on correction and repetition for me is what will harmonize the movements between the three athletes (A2)*.”

As regards professional support, the team had two coaches (*sensei*) who helped with the technical and tactical components, correcting the movements and supporting the kata composition. For the physical training, each athlete had support from different professionals (i.e., personal trainer) who was not specialized in exercise prescription for professional athletes.

### Complementary support

During the preparatory period, the team described partnerships with professionals in the fields of physical therapy, nutrition, and psychology, in which they were treated with no additional financial costs. Athletes A1 and A3 reported they had a few injuries during the process, and they received physical therapy support from a private clinic with no costs to treat the injuries and mitigate the risk of future ones. Nutrition counseling was reported as insufficient during the training process. Regarding psychological support, athletes A2 already had long-term assistance and athletes A1 and A3 had support from a psychologist from the Brazilian Olympic Committee close to the competition.

Neither the team nor the individual athletes had a sponsor for full-time training during the preparation. The athletes reported only specific financial support to move from one city to another during the classification competitions and to go to the Brazilian Olympic Center (Rio de Janeiro) before traveling to Lima, for the Pan American Games. The lack of financial support was pointed out by the athletes as one of the major negative factors during the preparation process. “*If we had better support from the country and if A1 and A2 did not need to work during the preparation, they would have more time to dedicated for improve their nutrition habits. The lack of time negatively influenced the preparation process (A3).” “A sponsorship at the Brazilian Team level would be a sponsor that stays with the athlete during the whole year. The athlete needs to pay for their own nutrition supplementation and facilities to perform the strength training (A2)*.” The athletes informed that the support from Brazilian Olympic Committee comes only after winning a medal in an international competition. “*I never understood well, you need to be the best to receive something, but how can you win if you do not have support?*”

### Training perception and further suggestions

Regarding the training process, the athletes described that they should have performed more tests before starting the training plan, and structured the session according to the components that needed to be improved. Athlete A3 describes that a baseline evaluation before starting the preparation process could help in planning the training sessions. “*First step, I would perform physical tests to see how we are doing, to have a baseline parameter. I would perform a kata evaluation to see how we are, if we remember the timing (movements) and if we are still aligned. From these first evaluations (physical and technical), develop the specific training of the modality and the physical components according to the initial. That is what I would think*.” Similarly, athlete A2 reported “*I think, I would perform tests, in order to know if someone on the team had some injuries or something like that. And I would build strength training based on the results*.” The positive aspect of the training planning was the technical-tactical preparation. The athletes recognized that they were performing high-quality technical-tactical components due to the coaches’ expertise and their long-time experience and successful results in the discipline. “*I think that the technical sessions were well structured. We performed the ‘kihon’ with the team and then with the coaches individually. I believe that worked out very well (A2)*.”

When asked about what they suggest for the preparation process, the athletes described that they would invest in factors such as improving the resting/recovery period during the process, improving the quality of the diet, and seek for psychological support. The psychological aspect was extensively explored by the athletes, highlighting the lacking of psychological support for the team during the preparation. “*Perhaps a psychologist as part of the staff of our team could have made a significant difference to achieve a better result (e.g., the gold medal)*.” According to the athletes, the Pan American Games can be considered one of the most important competitions for Brazilian kata athletes, therefore, the pressure during the preparation was very high, significantly requiring psychological preparation. “*It was highly mentally exhaustive (A2).” “Most of the time, it was not our body that was tired, it was more the mind because it was a lot, a lot of pressure (A3).” “I would not change the physical or technical preparation; however, I would be more relaxed during this process (A2)*.”

Moreover, the athletes pointed out the benefits of seeing each other frequently. The fact of being always together, every day for almost 1 year, was one more factor to justify the need for psychological support. The athletes compared their relationship to a marriage, “*We had argument and disagreement, but also needed to support each other (A1)*.” During this period, the support of a psychologist for the three athletes together, as a “couple therapy” could help in a better coexistence. “*If we have support from a psychologist since the beginning, as a therapist for couples, but instead for the team, we could certainly have been able to perform more in all the training sessions and probably perform better in the competition (A3)*.”

### Is there any question that was not asked, and you believe it should have been? Is there any aspect of your training that you would like to highlight about the preparation period?

When asked at the end of the interview if the athletes would like to add any point that was not addressed in the interview, athlete A1 point out “*The psychological part. I think is a very important part, that the athlete should focus a lot, especially high-performance athletes (A1)*.” The athlete A3 highlighted the importance of technical-tactical preparation. “*I think we did not comment about the level of instruction that we (the team) received. I believe that the level of instruction (from the coaches) was very high, and it favored a lot. Because there were teams stronger than us, but technically we were superior. So technical instruction is a crucial point, in preparation (A3)*.” The athlete A3 also highlights that kata is a modality whose strongest point is the technical factor and makes an analogy with soccer “*for soccer the important thing is not how perfect the pass is, it is important to make the ball come to the teammate*.” In the kata discipline, technical performance is a key element.

**FIGURE 1:**
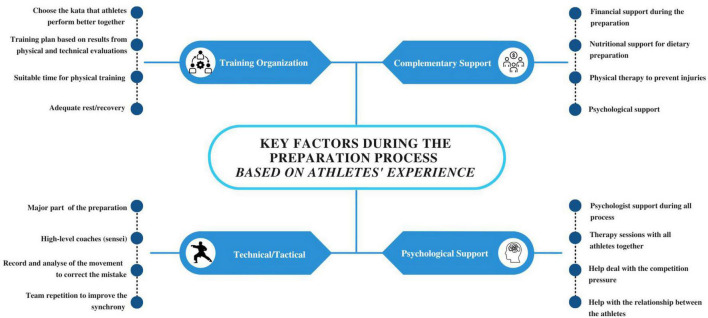
The athletes’ perception of the key factors during the preparation process for major kata competitions.

Figure 1 summarizes the key factors described by the athletes to improve the preparation process for major competitions for the kata team. According to their experience in the 2019 Pan American Games, it was possible to identify in the interview some recommendations about training organization, technical-tactical training, complementary support, and psychological support.

## Discussion

This present study aimed to describe the key factors involved in the preparation process of the Brazilian bronze medal kata team for the 2019 Pan American Games, focusing on the athletes’ perception. The main findings, according to the interview analysis were: (a) technical and tactical training took the major part of the preparation; (b) the high level of the coaches helped the team to reach the technical quality of the kata; (c) better psychological support during the preparation could improve the athletes’ performance during the training and competition; and (d) the lack of financial support compromised the commitment of the athletes during the training routine. The findings reinforce the extant literature while enhancing knowledge of the kata discipline in the context of the training and complementary support in which the article is grounded.

To plan an annual cycle is necessary to pay attention to principles related to the training process in order to focus on sport-specific needs to ensure optimal preparation for competition. Regarding the training process, the main aim is to *induce physiological adaptation and maximize performance at a specific point in time (the main competition)*. Each sport had a specific dominant factor to be training, therefore, the phases of preparation (general and specific) can be distinctive, the suggestion that non-specific training (e.g., general physical conditioning) can be introduced initially, and more specific tactical and technical training occurring more frequently when the main competition approaches (Plisk and Stone, 2003; Bompa and Buzzichelli, 2019). This concept has been endorsed previously in the periodization of mix-martial arts (James et al., 2013), and supported by the Brazilian kata team. This underpins the athletes’ description in the interview that during the training process, the technical and tactical components took a major part, and the high level of the coaches make a difference in improving the kata performance during the competition. The athletes did not deliberate adequate time for physical training during the process, and the restricted period for physical training could be related to a lack of time to fit the training into the daily routine. Even with some limitations to performing the physical training, the athletes recognized the importance of the physical component in the final performance. Recently, a scoping review showed that kata practice can improve cardiorespiratory fitness (Lassalvia et al., 2022), thus, it is possible to speculate that the physical components of the athletes were well-stimulated with the technical and physical session, even though the physical session was in lower proportion.

An important aspect mentioned by the athletes during the interview was financial support. Two athletes (A1 and A2) reported having jobs to support their affairs, training (e.g., gym memberships), and nutritional supplementation. Also, the lack of financial support led the athletes to spend time on the job (outside the sports context), affecting the time to perform physical training. Naturally, time spent on work obligations could affect the training plan in daily and weekly cycles, which may negatively affect the potential of load adaptation and the quality and speed of recovery. Indeed, financial support is one of the big issues among high-level athletes. A recent study interviewed 20 retired Olympic athletes from different sports modalities representing six different countries (Japan, Mexico, Portugal, Singapore, Republic of Korea, and the UK), and demonstrated that all athletes experienced financial challenges and struggles. Asking about the coping strategies to overcome their financial challenges, the athletes reported part-time jobs and training focused on medals and high ranks, which is likely to result in organizational financial support (Hong and Fraser, 2021). Similarly, Brazilian financial support for athletes is based on their results (medals) in competitions. The “*Bolsa Atleta*” is a program that directs resources from the Brazilian Ministry of Sports to athletes who reached the podium in national and international competitions (Brasil, 2004). However, as pointed out by athlete A3, “*is necessary to win to receive the financial resource, and how you can win with you do not have the adequate resource to improve your performance?*”

In addition to the training plan, the readiness of the athlete or the team for competition involves a complex interaction of factors such as well-planned nutrition, management of injuries, and psychological responses (Bompa and Buzzichelli, 2019). The athletes reported a lack of support on nutritional and psychological aspects throughout the process. The psychological aspect, however, was the most mentioned during the interview. The psychological demands in high-level sports settings can be related to the elevated competitive pressure, high training load, increased performance expectations, and numerous transitional events that can represent a significant threat to athletes’ mental health (Henriksen et al., 2020). The pressure to perform a high-quality kata routine could trigger mental health impairment in the athletes. Indeed, all athletes highlighted the necessity of a psychological follow-up during the process, to minimize the demand of the training and the stress of the competition. The recent literature has discussed and recommended strategies during the preparation process (pre-games phase) to take care of mental health issues in athletes, and promote self-care for the athletic population as well as coaches and staff to reduce unnecessary distress (Henriksen et al., 2020). Thus, the present study highlights the importance of mental health intervention in the kata discipline as well.

One limitation of our study is the assessment of one kata team only. The case study approach is limited to a specific population, thus the results should be interpreted with caution and cannot be extrapolated to other contexts and other countries/teams. The present study assessed three athletes. A previous case study in sport science can vary from one athlete (Hare et al., 2008) to a specific group of athletes (Kerr and Grange, 2009) showing important findings. Besides the limitations, to our knowledge, this is the first study describing the preparation process of a medal kata team for a major competition. Additionally, the interview presented a retrospective nature and was performed before the games could limit the memory of the events (Bryant et al., 2006). However, as the 2019 Pan American Games was one of the most important competitions in those athletes’ careers, it is likely that they accurately recalled their preparation process. Finally, we did not incorporate any statistical analytical procedure to determine intra- and inter-coder reliability as we kept the qualitative nature of the coding/theming process. Future investigations could add both a qualitative and a quantitative procedure for coding reliability to improve the communicability of the coding frame.

## Conclusion

In conclusion, based on the athletes’ perception, the positive factor during the process of preparation for a major competition was the high amount of time focused on technical-tactical training. Even with some limitations to performing the physical training, the athletes recognized the importance of the physical component in the final performance. As areas raised by the athletes need more significant improvements in the future, financial and psychological support could have helped the team to reach a better result (gold medal) by attenuating the training distress.

## Data availability statement

The raw data supporting the conclusions of this article will be made available by the authors, without undue reservation.

## Ethics statement

The studies involving human participants were reviewed and approved by Unicentro (number: 3.594.757). The patients/participants provided their written informed consent to participate in this study.

## Author contributions

ACP, CL, and DS: article conceptualization. ACP and CL: data selection. ACP, CL, and IM: data analysis. ACP, CL, IM, JC, and DS: drafted manuscript. All authors critically revised the manuscript, contributed to the article, and approved the submitted version.
